# Analysis of epilepsy‐associated variants in *HCN3* – Functional implications and clinical observations

**DOI:** 10.1002/epi4.13049

**Published:** 2024-10-03

**Authors:** Peiwei Zhao, Hongbo Xiong, Gunagtao Kuang, Chen Sun, Xiankai Zhang, Yufeng Huang, Sukun Luo, Lei Zhang, Jun Jiang, Xuelian He

**Affiliations:** ^1^ Precision Medical Center, Wuhan Children's Hospital (Wuhan Maternal and Child Healthcare Hospital), Tongji Medical College Huazhong University of Science & Technology Wuhan China; ^2^ Department of Cardiology, Zhongnan Hospital Wuhan University Wuhan China; ^3^ Department of Neuroelectrophysiology, Wuhan Children's Hospital (Wuhan Maternal and Child Healthcare Hospital), Tongji Medical College Huazhong University of Science & Technology Wuhan China; ^4^ Maternal Health Care Department, Wuhan Children’s Hospital (Wuhan Maternal and Child Healthcare Hospital), Tongji Medical College Huazhong University of Science & Technology Wuhan China; ^5^ Clinical Medical Research Center for Birth Defect Prevention and Treatmentin Wuhan Wuhan Children’s Hospital (Wuhan Maternal and Child Healthcare Hospital) Tongji Medical College, Huazhong University of Science & Technology Wuhan China

**Keywords:** current density, epilepsy, HCN ion channels, *HCN3*

## Abstract

**Objective:**

This case study investigates the role of hyperpolarization‐activated, cyclic nucleotide‐gated (HCN) ion channels, which are integral membrane proteins crucial for regulating neuronal excitability. HCN channels are composed of four subunits (HCN1‐4), with *HCN1*, *HCN2*, and *HCN4* previously linked to epilepsy. However, the role of the *HCN3* in epileptogenesis remains underexplored.

**Methods:**

We recruited a cohort of 298 epilepsy patients to screen for genetic variants in the *HCN3* (NM_020897.3) using Sanger sequencing. We identified rare variants and conducted functional assays to evaluate their pathogenicity.

**Results:**

We identified three rare heterozygous variants in *HCN3*: c.1370G > A (R457H), c.1982G > A (R661Q), and c.1982G > A(P630L). In vitro functional analyses demonstrated that these variants affected the expression level of *HCN3* protein without altering its membrane localization. Whole‐cell voltage‐clamp experiments showed that two variants (R457H and R661Q) significantly reduced current density in cells, while P630L has no effect on ion channel current.

**Significance:**

Our findings suggest that the identified *HCN3* genetic variants disrupt HCN ion channel function, highlighting *HCN3* as a novel candidate gene involved in epileptic disorders. This expands the genetic landscape of epilepsy and provides new insights into its molecular underpinnings.

**Plain Language Summary:**

Epilepsy is a brain disease that can be caused by mutations in specific genes. We found three rare variants in HCN3 gene in 298 patients with epilepsy, and two of the three mutations could be pathogenic and cause epilepsy and another one is single‐nucleotide polymorphism, which could have no effect and no contribution to the development of epilepsy.

**Key points**

- This is a report of two patients with epilepsy harboring *HCN3* heterozygous variants.
- In vitro functional analysis demonstrated that these two *HCN3* genetic variants decreased current densities, which could potentially affect neuronal excitability.
- The outcome of patients with *HCN3* variants is good.
- *HCN3* variant should be considered in patients with epilepsy.

## 1 INTRODUCTION

Hyperpolarization‐activated, cyclic nucleotide‐gated (HCN) ion channels are a unique class of voltage‐gated ion channels characterized by their dual activation through membrane potentials and cyclic adenosine monophosphate (cAMP) binding.^1^ These channels are crucial for modulating membrane excitability, as they conduct an inward, depolarizing current named If (“funny” current) in the heart and Ih (“hyperpolarization‐activated” current) in the brain.^2^, ^3^ In the nervous system, HCN channels perform multiple regulatory functions, including modulating neuronal excitability, integrating dendritic inputs, and facilitating presynaptic neurotransmitter release.^1^ HCN channels are encoded by four distinct genes (*HCN1‐4*), each corresponding to one of the four subtypes. While all four subtypes are expressed in the brain, their distribution varies: HCN1 is predominant in the neocortex and hippocampus; HCN2 is most abundant in the thalamus; HCN3 exhibits a diffuse expression pattern; and HCN4 is primarily localized in thalamic relay neurons.^3^, ^4^ Structurally, HCN channels are tetramers, with each monomer comprising six transmembrane segments (S1‐S6) and cytosolic N‐terminal and C‐terminal domains. The C‐terminal domain consists of the C‐linker and the cyclic nucleotide‐binding domain (CNBD), which regulates the response to cAMP.^5^ The sensitivity of HCN channels to cAMP varies among the subtypes. HCN2 and HCN4 are highly sensitive to cAMP, which enhances their voltage‐dependent activation. HCN1 has a moderate response, and HCN3 is either insensitive or inhibited by cAMP.^4^, ^6^, ^7^, ^8^ This differential sensitivity allows for precise regulation of their function in various physiological contexts.

Previous studies in animal models and humans have indicated that aberrations in ion channel expression, assembly, trafficking, and posttranslational modifications contribute to epilepsy.^9^, ^10^ Genetic variants in the *HCN1*, *HCN2*, and *HCN4* have been identified in patients with various forms of epilepsy, including febrile seizures, temporal lobe epilepsy, absence epilepsy, and epileptic encephalopathy.^11^, ^12^, ^13^, ^14^, ^15^ Although two non‐synonymous variants in *HCN* have been described in epilepsy patients,^16^ the role of *HCN3* in epileptogenesis remains poorly understood. Evaluating pathogenic variants in genes with high mutational tolerance, like *HCN3*, poses significant challenges in genetic research. The presence of numerous non‐pathogenic or low‐penetrance variants in large population databases such as gnomAD complicates the evaluation of *HCN3*'s potential pathogenic variants. This study aims to address these challenges by investigating the pathogenicity of novel *HCN3* variants identified in epilepsy patients. We adopted a focused approach to assess the functional impacts of these variants, acknowledging that traditional methods like whole‐exome sequencing (WES) or large epilepsy panels may miss subtle functional disruptions in genes with high mutational tolerance. Through detailed functional assays, we aim to elucidate the potential role of *HCN3* in epilepsy and provide a framework for evaluating variants in similar genes.

Here, we identified three novel *HCN3* variants, R457H, P630L and R661Q, through targeted genetic screening of 298 epilepsy patients using Sanger sequencing. Comprehensive in vitro functional assays demonstrated that these variants significantly affect channel function and stability, suggesting a potential role for *HCN3* in the development of epilepsy. Our findings underscore the importance of functional studies in validating the pathogenicity of variants in highly tolerant genes, advancing our understanding of their contributions to epilepsy.

## 2 MATERIALS AND METHODS

### 2.1 Study subjects

A total of 298 patients (167 males and 131 females) with clinically diagnosed epilepsy was recruited from Wuhan Children's Hospital, Tongji Medical College, Huazhong University of Science & Technology, between July 2013 and April 2019. Within this cohort, three unrelated patients were identified as carriers of *HCN3* variants. This study was approved by the institutional review board of Wuhan Children's Hospital, Tongji Medical College, Huazhong University of Science & Technology, ensuring compliance with ethical standards and patient consent protocols (2020R006‐E04).

### 2.2 Genetic analysis

Blood samples were collected from the probands and their parents. Genomic DNA was extracted from peripheral blood using the MicroElute Genomic DNA Kit (OMEGA Bio‐tek) according to the manufacturer's instructions. For RNA extraction, total RNA was extracted using Trizol reagent (Invitrogen). To identify and analyze genetic variants, Sanger sequencing was performed. The resulting data were compared against databases including the 1000 Genomes Project, dbSNP, gnomAD and ExAC. Variants were annotated and assessed for pathogenicity using the OMIM, HGMD, and ClinVar databases, adhering to the American College of Medical Genetics and Genomics (ACMG) guidelines. All *HCN3* variants identified in this study were described using the transcript reference sequence NM_020897.3. For patients with identified *HCN3* variants, whole‐exome sequencing (WES) was performed to screen for pathogenic variants in other known epilepsy‐related genes.

### 2.3 *HCN3* plasmid construction and cell transfection

Wild‐type *HCN3* fragment was amplified using *fast‐Pfu* enzyme, with human neural cell cDNA as a template; The amplified *HCN3* fragment was connected to the pCMV.2b vector to construct a wild‐type expression plasmid (WT‐*HCN3*). Site‐directed mutagenesis was performed to introduce a series of mutations (R457H, R661Q and P630L) using specific primers for linear amplification, followed by DpnI digestion of methylated DNA.^16^ All expression plasmids carry the FLAG tag at the C‐terminus, and all positive clones were verified by Sanger sequencing. HEK293T cells were grown in DMEM supplemented with 10% fetal bovine serum (Gibco, Thermo Fisher Scientific) and transfected with 2 μg plasmids (pCMV2b, WT, R457H, P630L, and R661Q) in 6‐well plates using Lipofectamine 3000 (Invitrogen) according to the manufacturer's instructions.

### 2.4 Western blot

Cells were lysed in 1% NP‐40 lysis buffer (50 mM Tris, pH 7.4, 150 mM NaCl, 1 mM EDTA, 1% NP‐40, and 0.5% sodium deoxycholate) for 30 min on ice and then centrifugated at 12 000 rpm for 10 min at 4°C. Cell membrane protein was extracted using Mem‐PER™ Plus membrane protein extract Kit (Thermo Fisher Scientific). Protein concentration was determined by BCA assay (Thermo Fisher Scientific), and 10 μg total protein was separated by 12% SDS‐PAGE and subsequently transferred to PVDF membrane. After blocking by 2.5% BSA, membranes were probed with the following antibodies: anti‐Flag (Proteintech, 66 008–4), anti‐Na,K‐ATPase Antibody(Cell Signaling Technology, #3101), and anti‐GAPDH (Proteintech, 60 004‐1‐Ig). Bound antibodies were detected using appropriate HRP‐conjugated secondary antibodies (Southern Biotech) and enhanced chemiluminescence (ECL, Thermo Fisher Scientific).

### 2.5 Electrophysiological studies

Whole‐cell voltage‐clamp recordings of *I* were performed as described previously.^17^, ^18^ The transfected HEK 293 T cells with wild‐type or mutant HCN3 plasmids were selected for whole‐cell voltage clamping recording of ion channel current. Currents were recorded in whole‐cell configuration using a Axopatch 700B amplifier and pClamp10.4 software. Analysis was done offline with Origin 8.0 software. Borosilicate glass pipettes for recording had a resistance of 2–4 megaohms when filled with intracellular (pipette) solution which contained 130 mM KCl, 10 mM NaCl, 0.5 mM MgCl, 1 mM EGTA, 5 mM HEPES, 3 mM Mg‐ATP, and 0.5 mM Na‐GTP; and pH adjusted to 7.4 with KOH. The extracellular solution contained 110 mM NaCl, 30 mM KCl, 1.8 mM CaCl_2_, 0.5 mM MgCl_2_, and 5 mM HEPES, pH adjusted to 7.4 with NaOH. The steady‐state activation of HCN3 is achieved through hyperpolarization voltage activation, with a step size of 10 mV between −140 mV and −20 mV, maintained at a clamp voltage of −40 mV every 3.6 s (pulse interval of 20 s), and a final step size of −140 mV every 0.5 s. Ih is determined by dividing the current across the test pulse (pA) by a single capacitor (pF).

### 2.6 Protein 3D modeling and structural analysis

The molecular model of wild‐type HCN3 was constructed using cryo‐electron microscopy data of human HCN3 (PDB ID: 8IO3). This wild‐type HCN3 3D model served as the template for modeling the R457H variant. The 3D models were graphically inspected using ChimeraX (UCSF, version 1.8) to analyze the structural impact of the variants on HCN3. To assess the effect on structural stability and flexibility, the tools mCSM and DynaMut2 (https://biosig.lab.uq.edu.au/tools) were utilized to calculate the Gibbs free energy changes (ΔΔG) and vibrational entropy energy differences between wild‐type HCN3 and the variant.

## 3 RESULTS

### 3.1 Case description

Patient 1 was a 5‐year‐old girl, the second child of healthy and unrelated Chinese parents. She was referred to our hospital due to refractory epilepsy at the age of 1 year and 7 months. She was born at term following an uneventful pregnancy and exhibited normal initial development. Her family history was unremarkable for developmental or epileptic disorders. The first epileptic seizure occurred at the age of 10 months, characterized by clonic seizures with eyes deviation and mouth corner chewing, lasting about 10 s. These seizures typically occurred while awake, and the child appeared drowsy afterward. No initial treatment was administered. Two weeks later, she presented new episodes of seizure with longer durations (60 s) and predominant tonic contraction of the left limb. Levetiracetam (30 mg/kg, bid) was prescribed but proved ineffective. The seizures progressed into a complex type, marked by binocular upward gaze, head backward extension, and hyperextension of the head and upper limbs. An electroencephalogram (EEG) showed hypsarrhythmia. Prednisone (1.0 mg/kg, bid) was added to the levetiracetam regimen, leading to a temporary remission of seizures. However, after 1 month, the seizures recurred in clusters of 5–6 per day, featuring head nodding followed by bilateral limb jerks lasting several seconds. Vigabatrin (125 mg, bid) introduction relieved the convulsions initially, but the seizures then changed to blank stares. Increasing the vigabatrin dosage worsened the head‐nodding convulsions, leading to its gradual reduction and discontinuation. Despite the combined therapy of levetiracetam (30 mg/kg, bid), prednisone (1.5 mg/kg in the morning, 1.0 mg/kg in the evening), and clonazepam (0.05 mg/kg nightly), the seizures persisted. She experienced 6–8 episodes of head nodding daily, each episode comprising 10–45 nods, primarily occurring after waking. Consequently, the patient was referred to our hospital at the age of 1 year and 7 months. On admission, the EEG showed multifocal abnormalities, especially in the bilateral frontal, occipital, and left temporal regions, with intensified epileptic discharge during sleep. Brain MRI examination appeared normal. After informed consent was obtained, treatment with adrenocorticotropic hormone (ACTH) at a dose of 150 U/m^2^ body surface area (BSA) was initiated, alongside vitamin B6. After 2 weeks of treatment, the patient's seizure decreased, and by the third week, the epileptic symptoms had disappeared. The ACTH treatment was weaned by the fourth week. Upon discharge, the patient was prescribed prednisone (2.0 mg/kg, qd), with the dosage gradually reduced by 2.5 mg per week, along with maintenance therapy of levetiracetam (30 mg/kg, bid), and clonazepam (0.05 mg/kg, nightly). No seizures recurred over more than 2 years of follow‐up.

Patient 2, a 1‐year 5‐month old boy, was born to a non‐consanguineous couple with a history of HIE disease. He was referred to our hospital due to nodding seizures for 2 months. The electroencephalogram indicated hypsarrhythmia, and the diagnosis of infantile spasms was considered. Topiramate and sodium valproate were used without significance. Physical examination including routine blood tests and liver and kidney function tests revealed no abnormalities. The patient has seizures every day after admission, 3–5 times a day, 7–8 groups a time, mostly with noticeable attacks after waking up. After admission, clonazepam (0.125 mg/kg, bid), topiramate (2.5 mg/kg, bid), and sodium valproate (15 mg/kg, bid) were used for antiseizure medication treatment and no seizures within a month. One month later, the patient showed similar symptoms and was readmitted to our hospital. Prednisolone (1.0 mg/kg, 4 times per day) was added to control seizures. This patient had a good treatment effect and has remained seizure‐free for more than 2 years following later treatment.

Patient 3, a 4‐year‐old boy, was born to a non‐consanguineous couple. He was delivered at term via lower segment cesarean section after an unremarkable pregnancy. At the age of 6 months, he started to have seizures characterized by clusters of episodic inversion and staring of eyes with head nodding. Each cluster comprised 20–30 episodes, lasted 2–3 min, and occurred 3–5 times per day. Physical examination revealed no abnormalities, and routine blood tests along with liver and kidney function tests were normal. An EEG showed hypsarrhythmia, while brain MRI findings were normal. Based on these observations, a diagnosis of infantile epileptic spasms was considered. After obtaining informed consent from the patient's parents, treatment with prednisolone (1.0 mg/kg, 4 times per day) was administered. On the second day after prednisolone treatment, there was a notable decrease in the frequency of seizures. By the third day, the seizures had completely ceased. The patient has remained seizure‐free for more than 2 years following the initiation of treatment.

### 3.2 Genetic analysis

**FIGURE 1:**
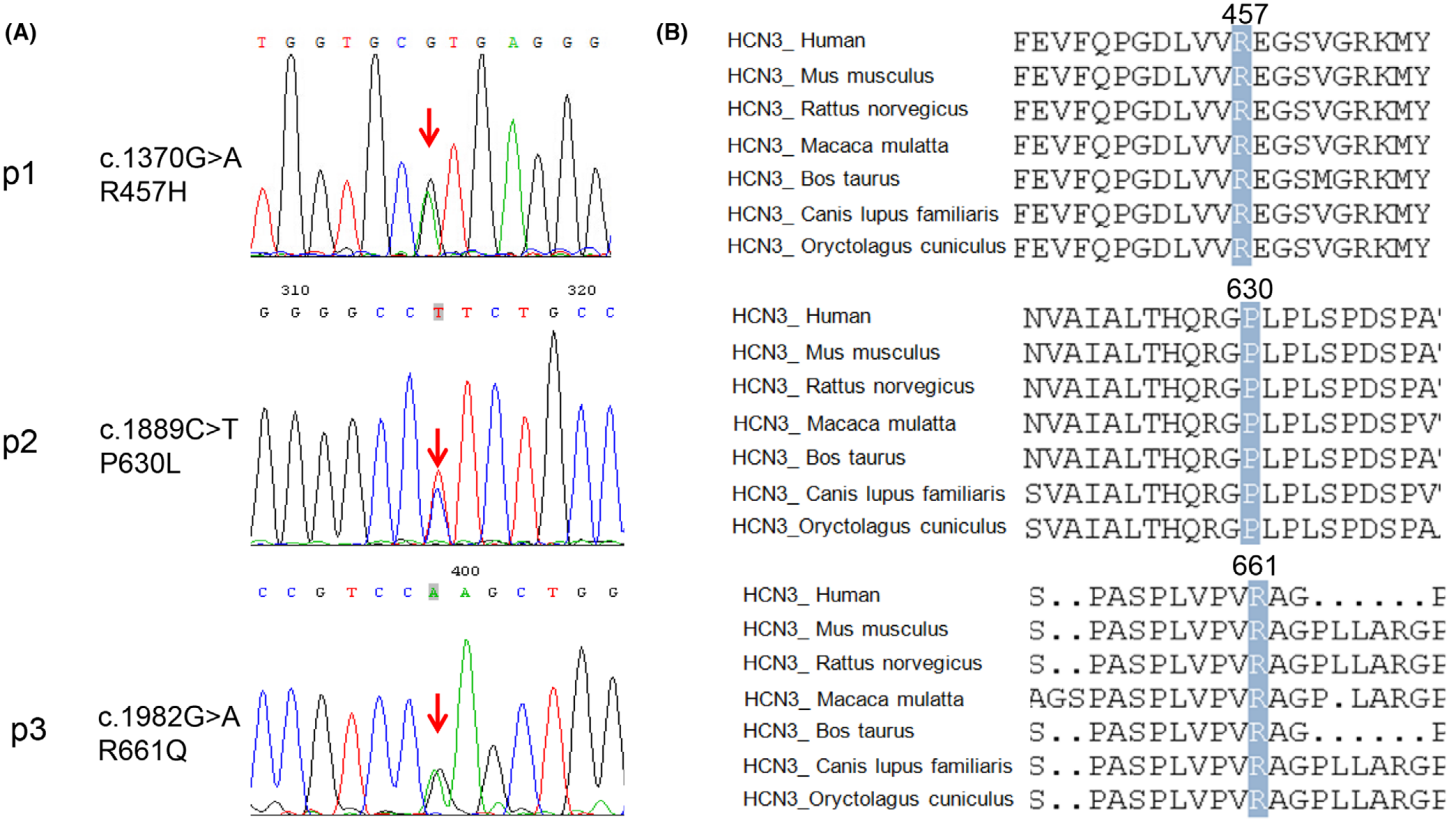
HCN3 mutations were detected in three patients. (A) Sanger sequencing of three *HCN3* variants in the family of this study and conservation analysis of HCN3 protein among different species. The positions of the variants at amino acids (R457, P630 and R661) were indicated and were highly conserved throughout all indicated species. (B) Schematic of the HCN3 channel, showing the location of the amino acids affected by the missense mutations identified in this study.

We identified two unrelated individuals with heterozygous variants in *HCN3* (NM_020897.3) through Sanger sequencing: c.1370G > A (p.R457H) in Patient 1, c.1889 C > T(p.P630L) in Patient 2 and c.1982G > A (p.R661Q) in Patient 3 (Figure 1A). All the variants were confirmed as de novo, with no presence in the respective parental genomes (PS2). The R457H is present 42 times in gnomAD v4.1.0 database with a minor frequency (MAF) of 2.60e‐5, and the R661Q variant is listed 9 times in 1 601 438 alleles with a MAF of 5.62e‐6. The low MAF for both variants in large population databases supports their rarity (PM2_Supporting).

The R457H variant has a Combined Annotation Dependent Depletion (CADD) score of 33, indicating a high likelihood of deleteriousness. It is predicted to be deleterious by different prediction tools: PolyPhen2 (probably damaging, score 0.999), SIFT (deleterious, score 0.001), MutationTaster (disease‐causing, score 1), and PROVEAN (damaging, score − 4.54), and has a GERP score of 5.46, indicating strong evolutionary conservation.

P630L variant in Patient 2 is present 8 times in gnomAD v4.1.0 database in East Asian population with a minor frequency (MAF) of 4.01e‐4, and the frequency in local database is 7.99e‐4, but the frequency is higher in European and Latino American population.

Similarly, the R661Q variant identified in Patient 3 has a CADD score of 22.1 and is predicted to be deleterious by PolyPhen2 (probably damaging, score 0.974), with a GERP score of 4.91, suggesting significant conservation. In silico models predict that these changes would disrupt the protein's structure and function (PP3). Both alanine at position 457 and arginine at position 661 are highly conversed among different species (Figure 1B). These two variants were classified as likely pathogenic according to the ACMG guidelines for variant classification, fulfilling criteria PS2 (de novo), PM2_Supporting (rarity in population databases), and PP3 (in silico pathogenicity predictions). To ensure there were no additional genetic variants contributing to epilepsy in these patients, we performed WES. The WES results confirmed that no other pathogenic or potentially pathogenic variants were present in known epilepsy‐related genes.

### 3.3 Functional analyses of the identified *HCN3* variants

In order to evaluate the effects of these two variants, we engineered and transfected 293 T cells with either R457H‐HCN3, R661Q‐HCN3, WT‐HCN3, or pCMV.2B (empty plasmid). P630L was reported by Tu et al.^16^ in the postmortem sample from a SUDEP patient, and this variant lacks relevant functional investigation. In addition, we also detected this variant in a patient with epilepsy in our study, who was excluded as he suffered hypoxia at birth and was diagnosed with hypoxic–ischemic encephalopathy. To investigate the effect of P630L, P630L‐HCN3 was constructed and examined in current study.

**FIGURE 2:**
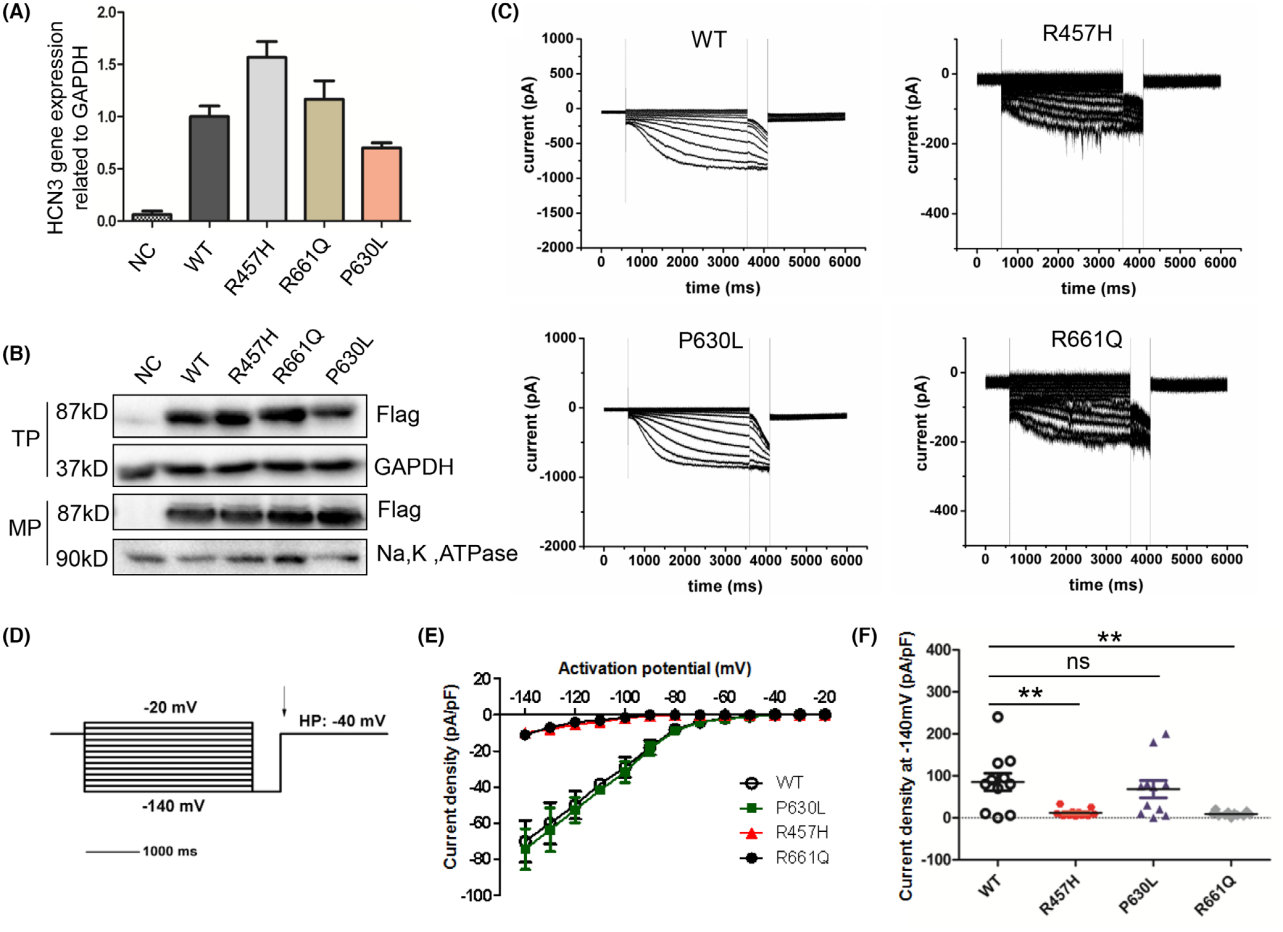
In vitro expression and patch‐clamp analysis of the functional effects of the HCN3 mutations. (A) The mRNA expression levels of HCN3 in 293 T cells transfected with wild‐type and mutated plasmids. (B) The expression level of the variants compared with the wild‐type in total protein and in cell membrane protein. (C) Electrophysiological recordings of HCN3 channels expressed in HEK293 cells. Representative current traces of HEK293 cells expressing WT and mutant HCN3 channels. (D) The used pulse protocol as followings. The first step, an activation for 3.6 s at potentials between −140 and −20 mV were used to determine voltage‐dependent activation time constants. The second step, a pulse to −140 mV after the initial activations at various potentials (arrow) was used to determine the voltage‐dependent activation curves. (E) The current density (pA/pF) of ion channels of cells transfected withWT and different variants under different voltage conditions(−140 ~ −20 mV). (F) The peak current density of mutant cells and WT cells under the −140 mV voltage condition. **p < 0.05.

After quantifying the expression and intracellular distributions of these HCN genes and proteins, no significant change was observed, except for increasing expression of R457H mRNA (Figure 2A,B). To verify if these mutations modify functional properties of HCN3 channels, whole‐cell voltage‐clamp analysis was performed in 293 T cells.

For electrophysiological evaluation, cell clones were chosen with current densities of −80 to 30 picoamperes/picofarads at −140 mV. Figure 2C shows examples of typical whole‐cell HCN3 currents of the indicated subtype, recorded with the protocol displayed in Figure 2D. All four HCN3 clones induce stable voltage‐dependent inward cation currents with distinct activation kinetics.

By measuring the current density (pA/pF) of ion channels under different voltage conditions (−140 ~ −20 mV), we found that current densities of cells transfected with variants (R457H and R661Q) were significantly decreased compared with that of the cells with WT‐ and P630L‐HCN3 at −90 ~ −140 mV (Figure 2E). In addition, the peak current density under the −140 mV voltage was measured, similar results were obtained, with the peak current densities of R457H cells and R661Q cells being significantly decreased than that of WT‐HCN3 and P630L cells (Figure 2F). These data indicate that both R457H and R661Q decreased the current density, suggesting these two variants likely exerted loss‐of‐function effects on HCN1 channels, while P630L does not exert any effect.

**FIGURE 3:**
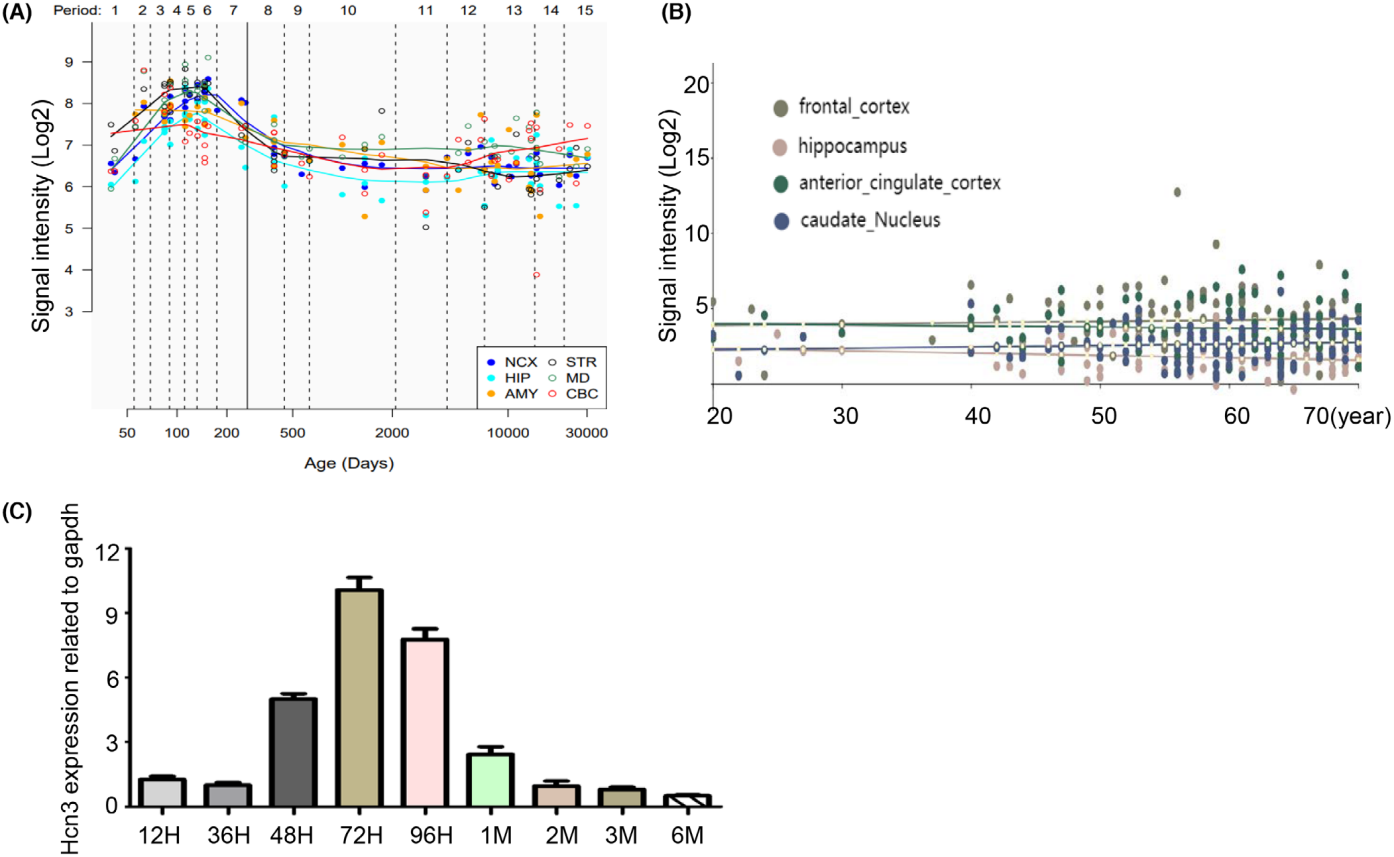
The expression levels of HCN3 at the HBT (human brain transcriptome) database and Brainexp database in the fetal period and after birth, respectively (A, B). The expression levels of HCN3 in zebrafish during the fertilized egg stage and after hatching (after 72 h) (C). H, hour; M: month.

In order to explain why these two patients with *HCN3* variants have better outcomes compared with patients with genetic variants in other *HCNs*, we analyzed the dynamic gene expression along the entire development and adulthood by using the HBT (Human Brain Transcriptome) database (https://hbatlas.org/) and Brainexp database (http://www.brainexp.org/). We found that the expression level of *HCN3* is relatively high in the fetal period, and it significantly decreases after birth (Figure 3A,B), and there is a similar trend in gene expression levels in zebrafish (Figure 3C). These findings are consistent with a previous study which showed that HCN3 expression was higher in young versus older animals and postulated that HCN3 likely does not contribute to alterations in Ih in older animals.^19^ Taken together, HCN3 may not be important for neuronal excitability in childhood and adulthood as in infancy, thus, the effect of genetic variants gradually disappear with age.

### 3.4 Structural impacts of identified pathogenic variants on the protein models of HCN3

**FIGURE 4:**
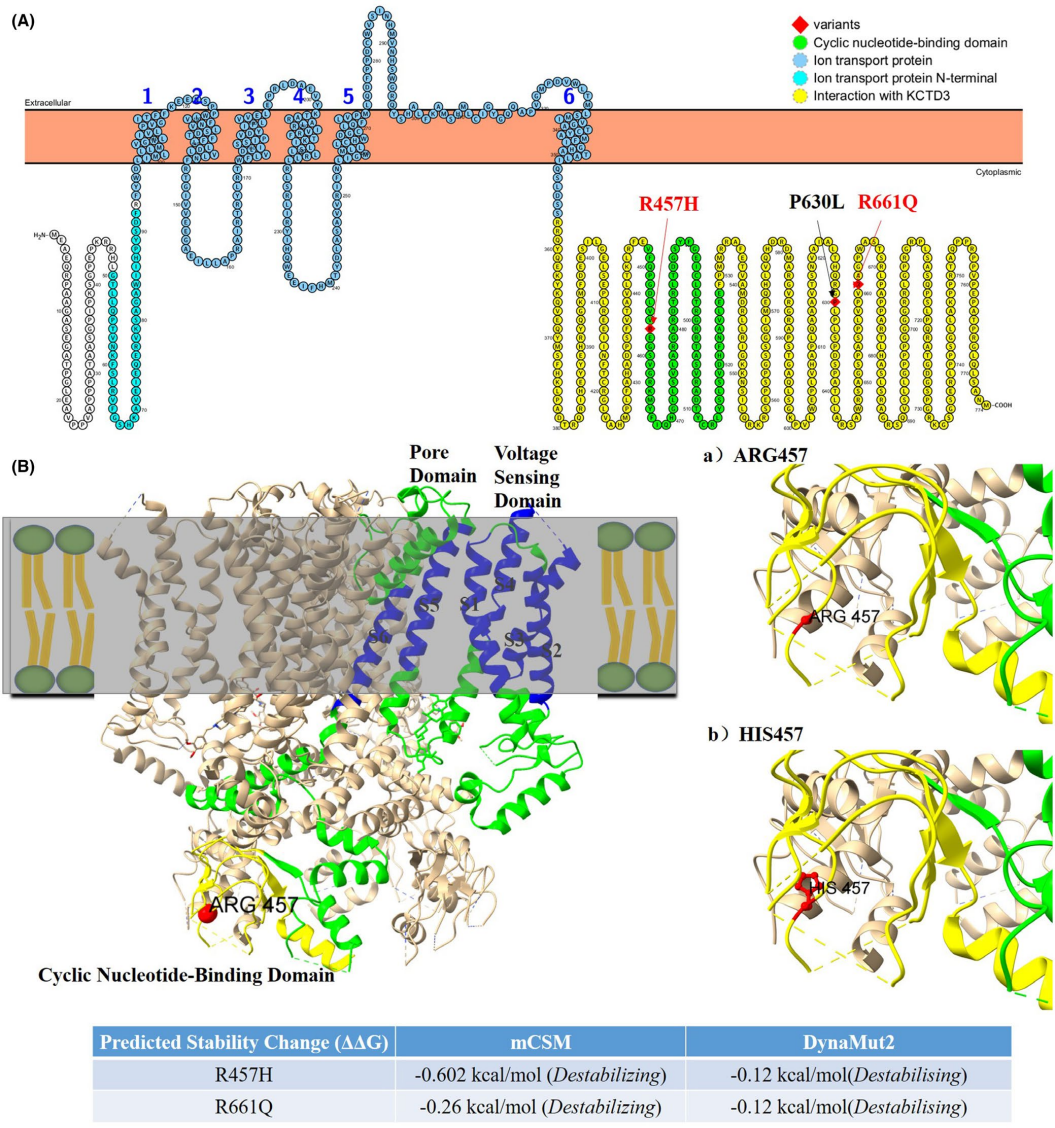
Membrane topology plot of HCN3 was generated using PROTTER, highlighting previously reported variants in black and currently identified variants in red. The plot shows the six transmembrane helices (S1–S6), numbered in the context of the ion transport domain depicted in blue. The cyclic nucleotide‐binding domain (CNBD) is shown in green and is located at the N‐terminus (A). 3D structural models of the HCN3 protein (PDB ID: 8IO3) are presented as ribbon structures, with the R457 residue highlighted as a ball. Part (a) illustrates the wild‐type HCN3 protein featuring the native Arg457, while part (b) displays the mutated HCN3 with His457 (B).

To investigate the structural impact of the identified R457H and R661Q variants, we developed both 2D and 3D models of the HCN3 channel (Figure 4). The R457H and R661Q variants are situated in the C‐terminal region, with R457 specifically located at the critical turns of the cyclic nucleotide‐binding domain (CNBD). This region is crucial for the channel's function as it modulates channel opening in response to cyclic nucleotides. In our structural analysis, we assessed the stability of the HCN3 channel by calculating the Gibbs free energy changes (ΔΔG) associated with each missense variant. These ΔΔG values provide insights into how each variant affects the protein's structural integrity and flexibility. R457H was modeled onto the HCN3 3D structure to visualize their impacts on the channel's conformation. R457H is located in the CNBD, and the substitution of arginine (a positively charged amino acid) with histidine (a less positively charged amino acid) is likely to alter the local conformation. Similarly, R661Q involves the replacement of arginine with glutamine, which could affect the overall stability. The ΔΔG values calculated using the mCSM and DynaMut2 tools revealed a decrease in stability for both variants, indicating that these changes could lead to altered channel function or decreased protein stability.

## 4 DISCUSSION

Epilepsy is a prevalent and severe neurological disease with a significant genetic component. Ion channel genes, in particular, are implicated in about 25% of genetics epilepsy.^9^ These inherited or sporadic genetic variants can alter membrane expression or the biophysical properties of ion channels, leading to epilepsy.^9^, ^20^ In recent years, an increasing number of variants in ion channel genes have been identified and characterized. These genetic and pathophysiological investigations have deepened our understanding of epileptogenesis and are instrumental in improving anticonvulsive therapy.

HCN ion channels, a unique class of ion channels, are involved in generating spontaneous rhythmic activity in cardiac pacemaker and neuronal cells by regulating cell membrane excitability.^1^, ^2^ While HCN1, HCN2, and HCN4 have well‐documented roles in determining resting membrane potential, dendritic integration, synaptic transmission, and action potential firing, the function of HCN3 has been less studied.^21^, ^22^ Furthermore, the evidence for variants in *HCN1*, *HCN2*, and *HCN4* in human epilepsy has rapidly grown in recent years.^11^, ^12^, ^13^, ^14^, ^15^ However, the association of *HCN3* variants with epilepsy has been scarcely explored. In this study, we identified three variants in the *HCN3* in individuals with epilepsy who responded well to treatment. These patients exhibited clonic seizures or infantile and epileptic spasms. In vitro functional analysis demonstrated that two *HCN3* variants led to decreased current densities, which could potentially affect neuronal excitability. Structural predictions for the HCN3 channel variants R457H and R661Q aligned well with clinical and electrophysiological findings. The R457H variant demonstrated impaired function, indicating a destabilized CNBD and disrupted cyclic nucleotide binding, supporting its potential pathogenicity. The R661Q variant decreased stability, suggesting a link to decreased current density. These findings suggest that *HCN3* could be a novel pathogenic gene implicated in the pathogenesis of epilepsy.

HCN channels are a family of four isoforms, each with distinct expression patterns and roles within the nervous system. HCN1 and HCN2 are the predominant isoforms in the brain, with HCN1 mainly expressed in the neocortex, hippocampus, cerebellar cortex and brainstem, and HCN2 largely found in the thalamus.^3^, ^23^ HCN3 has a more diffuse but low‐level expression throughout the nervous system, while HCN4's distribution complements that of HCN1, being expressed in areas where HCN1 is less prominent.^3^, ^24^ The expression, kinetics, and gating properties of HCN channels in the brain are influenced by several factors, including epileptic activity, age, interactions with various intracellular molecules or proteins such as cAMP, PIP2, p38‐MAPK, β‐subunits, filamin A, and TRIP8b.^1^, ^25^ For instance, Kanyshkova et al.^19^ reported that HCN3 expression was higher in younger animals compared to older ones, suggesting that HCN3 may not significantly contribute to alterations in Ih in older animals. The varying expression levels across different brain regions might partially explain the diverse physiological roles of Ih in these areas. In addition to expression levels and localization, small molecules like cAMP can modulate HCN channel properties in a subtype‐specific manner by directly binding to CNBD of the C‐terminal domain.^26^

Among the four HCN isoforms, *HCN3*, located on chromosome 1q22, is the least characterized and has the smallest protein size, with only 774 amino acids. Despite its less prominent expression and lower functional characterization, HCN3 is known to have distinct roles in the CNS.^4^ Stieglitz et al.^24^ demonstrated that HCN3 might alter the intrinsic activity of neurons in the amygdala complex or hippocampal neurons, potentially disrupting the proper processing of learned fear in HCN3^−/−^ mice. Lainez et al.^27^ further showed that while HCN3 contributes to the excitability of medium‐sized neurons, it does not play a significant role in either acute or chronic pain sensation. To date, there is no animal model for epilepsy specifically related to HCN3. However, Tu et al.^16^ reported two non‐synonymous variants (p.K69R and p.P630L) of *HCN3* in postmortem studies of individuals with epilepsy and sudden unexpected death in epilepsy (SUDEP). The functional properties and clinical phenotypes associated with these variants were inadequate, and other pathogenic genetic variants had not been ruled out by WES of other known epilepsy‐related genes.

In the current study, both identified pathogenic variants, R457H and R661Q, are located in the C‐terminal domain of the HCN3 channel. Functional study indicates that both variants lead to a loss‐of‐function for HCN3, similar to the effects observed with the I380F and A395G variants in *HCN1*.^28^ Xie et al.^28^ showed that both heterozygous I380F and A395G HCN1 channels exhibited a reduced current density compared to wild‐type channels, though the impact was less pronounced than that of the homozygous mutant HCN1 channels. In our study, we did not investigate the effects of the heterozygous state of HCN3, which represents a limitation that needs to be addressed in future research. The P630L variant, first reported by Tu et al.^16^ in the postmortem sample from a SUDEP patient in 2011, was proved to have no significant effect on the biological properties of HCN3. According to the gnomAD v4.1.0 database, this variant is present in 42 785 heterozygous individuals out of 1 613 936 alleles (frequency of 2.65e‐2) and 747 homozygotes, suggesting it is a common variant. Predictive tools such as Polyphen and PROVEAN classify this variant as benign and tolerable, respectively, indicating that it may represent a polymorphism change rather than a pathogenic mutation.

Following treatment with antiseizure medications, both patients in our study became seizure‐free. It is noteworthy that seizure relapse occurs in approximately 34% of epilepsy patients,^29^ and the expression level of epilepsy‐related genes is crucial for the prognosis of epilepsy.^30^ As mentioned above, HCN3 expression is higher in young versus older rats,^19^ and the expression patterns of HCN3 in zebrafish and human show a similar trend, with levels decreasing gradually with age.

Our study was initiated in 2014, following the discovery of *HCN1*'s role in epilepsy, which sparked our interest in investigating the involvement of *HCN3* in epilepsy due to its similarities to HCN channel subtypes. At that time, whole‐exome sequencing (WES) was not as accessible or cost‐effective, so we used Sanger sequencing, which was more feasible for the *HCN3*. Our main goal was to identify potential pathogenic variants in *HCN3* and assess their impact on neuronal function. After identifying *HCN3* variants, we planned to conduct further studies, including WES, to explore other genetic factors contributing to epilepsy in our cohort. This phased approach allowed us to systematically identify and validate significant genetic variants. Despite *HCN3*'s high mutational tolerance, we found that the R457H and R661Q variants had significant functional effects in our in vitro assays, suggesting their potential contribution to disease. Our study demonstrates the potential of targeted genetic screening in identifying pathogenic variants, even in genes with high mutational tolerance. This approach is particularly valuable in resource‐limited settings. By using broader screening methods like WES, we can enhance our understanding of the genetic landscape and identify further genetic factors contributing to epilepsy.

In conclusion, we report two novel heterozygous variants in the *HCN3* identified in two unrelated patients with epilepsy. Our study provides functional evidence supporting the pathogenicity of these variants, thereby underscoring the potential causal role of *HCN3* in epilepsy. However, additional cases and further functional studies are necessary to deepen our understanding of the precise roles of *HCN3* in the context of ion channel function and its contribution to epileptic pathophysiology.

### 4.1 Limitations

In vitro functional study, we evaluated the effects of cells transfected with WT, mutant, and empty plasmids. However, we did not include co‐transfections with WT and mutant constructs to mimic the heterozygous status found in patients. In addition, we observed that *HCN3* variants resulted in decreased current densities, which could potentially affect neuronal excitability. However, further study to examine the underlying mechanism was not conducted due to the budget constraints and technical limitations.

## AUTHOR CONTRIBUTIONS

Study concepts: Xuelian He, Peiwei Zhao, and Jun Jiang. Study design: Peiwei Zhao, Hongbo Xiong, and Yufeng Huang. Literature research: Peiwei Zhao, Xiankai Zhang, and Yufeng Huang. Clinical information collection: Guangtao Kuang, Chen Sun, Xiankai Zhang, and Jun Jiang. Experiment: Peiwei Zhao, Hongbo Xiong, and Sukun Luo. Data analysis/interpretation: Peiwei Zhao, Guangtao Kuang, Chen Sun, Lei Zhang, and Sukun Luo. Manuscript preparation: Xuelian He, Peiwei Zhao, Guangtao Kuang, and Chen Sun. Manuscript editing: Xuelian He and Jun Jiang. Manuscript final version approval: Xuelian He.

## FUNDING INFORMATION

This work was supported by the grants of Hubei Provincial Natural Science Foundation Project (No. 2023AFB893); Neurological Disease Clinical Medical Research Center (WK2014‐160); Construction Project of Clinical Medical Research Center for Neurodevelopmental Disorders in Children in Hubei Province (HST2020‐19); and Construction Project of Clinical Medical Research Center for Birth Defect Prevention and Treatment in Wuhan (WK[2023]123‐4).

## CONFLICT OF INTEREST STATEMENT

None of the authors has any conflict of interest to disclose. We confirm that we have read the Journal's position on issues involved in ethical publication and affirm that this report is consistent with those guidelines.

## ETHICS APPROVAL STATEMENT

This study has been approved by the institutional review board of Wuhan Children's Hospital, Tongji Medical College, Huazhong University of Science & Technology.

## PATIENT CONSENT STATEMENT

Written informed consent was obtained from the patients' parents.

## PERMISSION TO REPRODUCE MATERIAL FROM OTHER SOURCES

No reproduced material needed copyright is involved.

## CLINICAL TRIAL REGISTRATION

ChiCTR2000034358.

## Data Availability

The data underlying this article will be shared on reasonable request to the corresponding author.
